# Efficacy and safety of tiotropium combined with budesonide/formoterol in treating elderly patients with chronic obstructive pulmonary disease

**DOI:** 10.12669/pjms.41.7.11995

**Published:** 2025-07

**Authors:** Fan Sun, Dandan Hu, Wenbing Liu, Xiaowei Wang, Xueyun Liu, Tuya Wulan

**Affiliations:** 1 Fan Sun, Department of Cardiopulmonary Intensive Care Rehabilitation, The Third Affiliated Hospital of Zhejiang Chinese Medical University, Hangzhou, Zhejiang Province 310005, P.R. China; 2 Dandan Hu, Department of Pulmonary and Critical Care Medicine, The Third Affiliated Hospital of Zhejiang Chinese Medical University, Hangzhou, Zhejiang Province 310005, P.R. China; 3 Wenbing Liu, Department of Cardiopulmonary Intensive Care Rehabilitation, The Third Affiliated Hospital of Zhejiang Chinese Medical University, Hangzhou, Zhejiang Province 310005, P.R. China; 4 Xiaowei Wang, Department of Cardiopulmonary Intensive Care Rehabilitation, The Third Affiliated Hospital of Zhejiang Chinese Medical University, Hangzhou, Zhejiang Province 310005, P.R. China; 5 Xueyun Liu, Department of Cardiopulmonary Intensive Care Rehabilitation, The Third Affiliated Hospital of Zhejiang Chinese Medical University, Hangzhou, Zhejiang Province 310005, P.R. China; 6 Tuya Wulan, Department of Cardiopulmonary Intensive Care Rehabilitation, Zhejiang Rehabilitation Medical Center, Hangzhou, Zhejiang Province 310052, P.R. China

**Keywords:** Adverse reactions, Budesonide/Formoterol, COPD, Pulmonary function, Tiotropium

## Abstract

**Objective:**

This study aimed to assess the efficacy and safety of the combined tiotropium (TIO) and budesonide/formoterol (BUD/FORM) treatment in elderly patients with chronic obstructive pulmonary disease (COPD).

**Methods:**

Medical records of elderly patients with COPD who received treatment at the Third Affiliated Hospital of Zhejiang University of TCM from May 2023 to August 2024 were retrospectively analyzed. Elderly patients with COPD receiving TIO+BUD/FORM combination treatment (TIO+BUD/FORM group) and patients receiving TIO treatment alone were matched for age in a 1:1 ratio. The primary outcome of interest was the baseline change in pulmonary function at the 12 weeks follow-up. The secondary outcome was the incidence of adverse reactions.

**Results:**

This study included 114 patients. Post-treatment levels of forced vital capacity (FVC), FEV1 as a percentage of the predicted value (FEV1%pred), forced expiratory volume in one second (FEV1), and six-minute walk test (6MWT) in both groups increased compared to the baseline and were significantly higher in the TIO+BUD/FORM group compared to the TIO group (*P*<0.05). The treatment led to a significant decrease in the COPD Assessment Test (CAT) and levels of high-mobility group box 1 (HMGB1), as well as high-sensitive C-reactive protein (hs CRP) in both groups. These indexes were considerably lower in the TIO+BUD/FORM group than in the TIO group (*P*<0.05). The two groups had no difference in the rate of adverse reactions (*P*>0.05).

**Conclusions:**

Compared with TIO alone, TIO combined with BUD/FORM is more effective in improving pulmonary function in elderly patients with COPD without increasing adverse reactions.

## INTRODUCTION

Chronic obstructive pulmonary disease (COPD) is a lung condition that is more common in the elderly population. This condition is characterized by chronic inflammation that predominantly impacts the lung parenchyma and peripheral airways and results in irreversible and progressive airflow limitation that may eventually lead to pulmonary heart disease and respiratory failure.^1^–^3^ The pathogenesis of COPD is still unclear, and the treatment mainly focuses on relieving clinical symptoms, controlling inflammation, and reducing acute disease attacks.^3^,^4^

Tiotropium bromide, a commonly used medication for treating COPD, belongs to the category of long-term cholinergic receptor antagonists. It acts on bronchial smooth muscle and continuously inhibits tracheal contraction.^5^ However, since small airway inflammation is one of the main characteristics of COPD, simple bronchiectasis is rarely effective enough, and there is a need to suppress airway inflammatory reactions and alleviate respiratory edema.^5^,^6^ A fixed-dose combination of the corticosteroid budesonide (BUD) and the long-acting beta2-agonist formoterol (FORM) that is used as long-term maintenance treatment for COPD was shown to effectively dilate bronchial smooth muscle, activate adenylate cyclase, dilate the trachea, and reduce inflammation.^7^ Since inflammatory response plays an important role in the onset and progression of COPD, monitoring changes in inflammatory factors can accurately evaluate disease efficacy.^8^,^9^ High sensitivity C-reactive protein (hs-CRP) is an acute phase reactive protein that can abnormally rise during infections, injuries, and inflammations.^9^ High mobility group protein B1 (HMGB1) is a late-stage inflammatory factor that can promote the expression of multiple downstream inflammatory effectors, thereby participating in airway remodeling and inflammatory response.^8^

Current studies have demonstrated the effectiveness of TIO and BUD/FORM in treating COPD.^10^,^11^ However, evaluating the efficacy of the combined TIO+BUD/FORM treatment in specific patient subgroups, such as elderly individuals, is crucial for developing informed medical practices. The physiological state of elderly COPD patients is complex, and there are specific challenges in selecting treatment plans. This retrospective study aimed to evaluate the efficacy and safety of the combination of TIO and BUD/FORM in elderly patients with COPD to provide a more targeted and scientific basis for the clinical treatment of this vulnerable group.

## METHODS

This retrospective cohort study included medical records of elderly patients with COPD treated at the Third Affiliated Hospital of Zhejiang University of Chinese Medicine from May 2023 to August 2024. Elderly patients with COPD receiving treatment with TIO combined with BUD/FORM (TIO + BUD/FORM group) were matched for age with a cohort receiving TIO treatment alone (TIO group) in a 1:1 ratio.

### Ethical approval:

All procedures followed the ethical standards outlined in the 1964 Helsinki Declaration and its subsequent amendments. This study has been approved by the Ethics Review Committee of the Third Affiliated Hospital of Zhejiang University of Traditional Chinese Medicine (No. ZSLL-KY-2022-008-01), Date: April 25^th^, 2022. Due to the study’s retrospective nature, the Ethics Review Committee of the Third Affiliated Hospital of Zhejiang University of Traditional Chinese Medicine waived the requirement for individual informed consent. This study was conducted under applicable guidelines and regulations. Each patient’s data was anonymous and de-identified to maintain confidentiality.

### Inclusion criteria:

- Meet the diagnostic criteria for COPD.^12^
- Age ≥ 60 years old.
- Not taking glucocorticoids or bronchodilators within four weeks before admission.
- Complete medical records.

### Exclusion criteria:

- Patients with airflow limitation caused by lung cancer, active pulmonary tuberculosis, cystic pulmonary fibrosis, and bronchiectasis.
- Patients with concomitant pulmonary encephalopathy.
- Patients with gastrointestinal bleeding.
- Patients with coagulation dysfunction.

### Treatment protocols and collected indicators:

After admission, routine interventions such as fluid replacement, cough and phlegm reduction, anti-infection, and low-flow oxygen therapy were given. Patients in both groups were treated with TIO alone or in combination with BUD/FORM for 12 weeks.

### TIO regimen:

Patients received inhaled powder mist of TIO (specification: 18 μg; Jiangsu Zhengda Tianqing Pharmaceutical Group Co., Ltd. China) using an inhalation device (HandiAler) to inhale once a day at a dose of 18 μg/time.

### TIO+BUD/FORM regimen:

Patients received BUD/FORM (specification: 160 μg/4.5 μg/key, 60 suctions/vial; AstraZeneca, UK) using a HandiAler inhalation device based on the TIO therapy twice a day, one inhalation each time.

The following indicators were collected from patients before and after 12 weeks of treatment:

- Pulmonary function indicators include forced expiratory volume in one second (FEV1), FEV1/forced vital capacity (FVC), and FEV1 as a percentage of predicted value (FEV1% pred). The above indicators were measured using an MSIOS professional lung function tester (produced by JAEGER, Germany).
- Severity of illness and exercise endurance. The severity of the condition was evaluated using the COPD Assessment Test (CAT).^13^ The test consists of eight items, each scored on a scale of 0-5 points, with a total score of 0-40 points. A higher score indicated more severe clinical symptoms of the patient. Assessment of exercise endurance based on a 6-minute walk test (6MWT)^14^ that was conducted by measuring the longest distance a patient can walk within six minutes while walking back and forth in a 20-meter-long corridor. The test was performed twice with an interval of ≥ 2 hours, and the optimal value was recorded. This test aims to evaluate the patient’s exercise tolerance.
- Serum HMGB1 and hs CRP levels were measured by enzyme-linked immunosorbent assay. The reagent kit was purchased from Shanghai Beyotime, and the operating steps were strictly followed according to the instructions.
- Adverse reactions include constipation, dizziness, dry mouth, and palpitations.

### Statistical analysis:

All statistical analyses were conducted using IBM SPSS Statistics for Windows 20.0, Armonk, New York: IBM Corporation. Normal distribution data was described as mean ± standard deviation (SD), while non-normal distribution data was reported as median (interquartile range, IQR) unless otherwise specified. For normally distributed data, an independent sample t-test was used for inter-group comparison, and a paired t-test was used for intra-group comparison before and after the treatment. The Mann-Whitney U test was used for inter-group comparison of non-normally distributed data, and the Wilcoxon signed-rank test was used for intra-group comparison before and after the treatment. Categorical variables were reported as frequency and percentage and evaluated using the chi-square test or Fisher’s exact test as appropriate. *P*<0.05 was considered statistically significant.

## RESULTS

This study included 114 elderly patients with COPD. The age of the patients ranged from 60 to 91 years, with a median age of 73 (66-79) years. There were 63 males in the cohort, accounting for 55.3%. Participants (n=57) who received a combined TIO and BUD/FORM treatment were matched in a 1:1 ratio with patients who received TIO alone. As shown in Table-I, there was no significant difference in basic clinical characteristics such as age, sex, disease duration, body mass index (BMI), GOLD grading, smoking status, alcohol consumption, and underlying diseases between the two groups of patients (*P*>0.05).

**Table-I:** Comparison of Basic Clinical Characteristics between Two Groups.

Characteristics	TIO + BUD/FORM group (n=57)	TIO group (n=57)	Z/t/χ^2^	P
Age (years), M(P25/P75)	71 (66-78)	75 (68-81)	-1.608	0.108
Male (yes), n (%)	34 (57.6)	29 (50.8)	0.036	0.849
Disease duration (years), M(P25/P75)	5 (4-7)	5 (4-6)	-0.833	0.377
BMI (kg/m2), mean±SD	22.20±3.66	22.14±3.56	0.086	0.932
***GOLD classification, n (%)***				
Grade-1	4 (7.0)	3 (5.3)	2.248	0.522
Grade-2	13 (22.8)	10 (17.5)		
Grade-3	24 (42.1)	32 (56.1)		
Grade-4	16 (28.1)	12 (21.1)		
Smoking situation (Yes), n (%)	27 (47.4)	24 (42.1)	0.319	0.572
Alcohol consumption (Yes), n (%)	19 (33.3)	25 (43.9)	1.332	0.248
Coronary heart disease (yes), n (%)	16 (28.1)	18 (31.6)	0.168	0.682
Hypertension (Yes), n (%)	22 (38.6)	14 (24.6)	2.598	0.107
Diabetes (Yes), n (%)	12 (21.1)	11 (19.3)	0.054	0.815
Hyperlipidemia (Yes), n (%)	7 (12.3)	4 (7.0)	0.906	0.341

**Fig.1:**
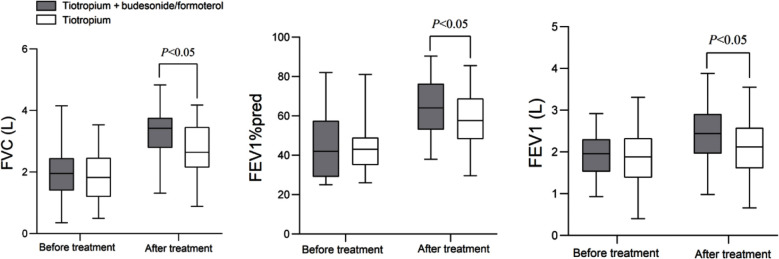
The changes in lung function indicators before and after treatment. Forced vital capacity (FVC); FEV1 as a percentage of the predicted value (FEV1%pred); Forced expiratory volume in 1 second (FEV1).

As demonstrated in Fig.1, before the treatment, there was no significant difference in FVC (1.95 (1.4-2.44) vs. 1.82 (1.2-2.46)), FEV1% (42 (29-57) vs. 43 (35-49)), and FEV1 (1.93±0.52 vs. 1.86±0.69) levels between the two groups (*P*>0.05). After 12 weeks of treatment, the levels of FVC (3.28±0.68 vs. 2.78±0.83), FEV1% (64.2±13.6 vs. 58.5±14.7), and FEV1 (2.46±0.66 vs. 2.08±0.71) in both groups increased compared to pretreatment and were significantly higher in the TIO+BUD/FORM group compared to the TIO group (*P*<0.05).

**Fig.2:**
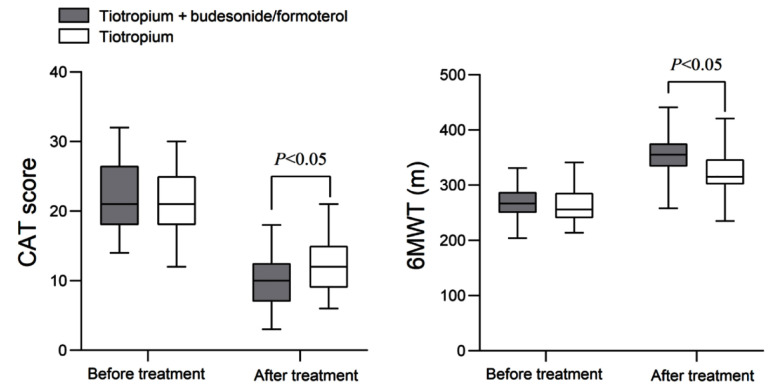
Changes in CAT and 6MWT before and after treatment in two groups of patients. COPD Assessment Test (CAT); Six-minute walk test (6MWT).

As demonstrated in Fig.2, pretreatment CAT scores and 6MWT of both groups were comparable (*P*>0.05). After treatment, the CAT scores of both groups decreased, while the 6MWT increased. The CAT score of the TIO+BUD/FORM group was lower, and the 6MWT was longer than that of the TIO group (*P*<0.05).

**Fig.3:**
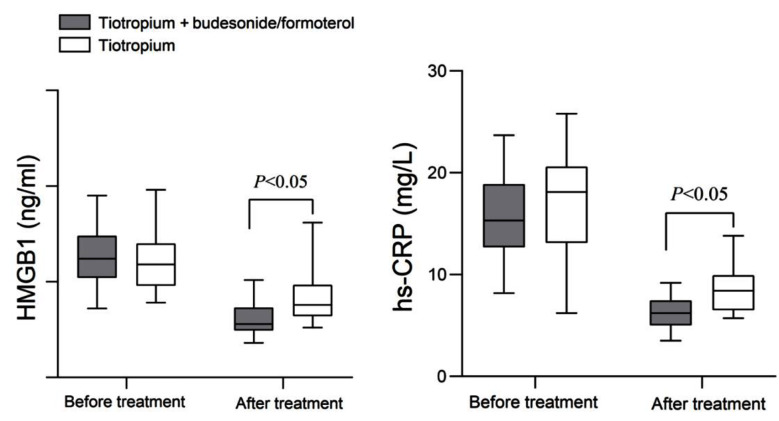
The changes in HMGB1 and hs CRP levels before and after treatment in two groups. High-mobility group box 1 (HMGB1); high-sensitive C-reactive protein (hs-CRP).

As demonstrated in Fig.3, there was no significant difference in serum HMGB1 and hs CRP levels between the two groups before the treatment (*P*>0.05). After treatment, both inflammatory indicators decreased in the two groups and were considerably lower in the TIO+BUD/FORM group than in the Tio group (*P*<0.05).

As summarized in Table-II, there was no significant intergroup difference in the incidence of adverse reactions (*P*>0.05). The reported adverse effects included two cases of constipation, two cases of dry mouth, and one case of palpitations in the TIO+BUD/FORM group, and one case of dizziness and one case of dry mouth in the TIO group (Table-II).

**Table-II:** Comparison of Adverse Reaction Rates between Two Groups.

Group	n	Constipation	Dizziness	Dry mouth	Palpitation	Total incidence rate
TIO+ BUD/FORM group	57	2 (3.5)	0 (0.0)	2 (3.5)	1 (1.8)	5 (8.8)
TIO group	57	0 (0.0)	1 (1.8)	1 (1.8)	0 (0.0)	2 (3.6)
*χ^2^*						1.370
*P*						0.435^b^

***Note:*** b, continuity correction.

## DISCUSSION

This study evaluated the efficacy and safety of the combined regimen of TIO and BUD/FORM in elderly patients with COPD. The results showed that, compared to TIO alone, combining TIO and BUD/FORM can more efficiently improve the pulmonary function of elderly patients with COPD, facilitates their physical recovery, and is associated with reduced levels of inflammatory markers.

This study showed that after 12 weeks of treatment, FVC, FEV1%, and FEV1 indexes increased significantly in both groups and were considerably higher in the TIO+BUD/FORM group. Previous studies have demonstrated that TIO is able to effectively dilate airway smooth muscle, increase airway diameter, and improve lung ventilation function,^5^ formoterol is a long-acting β_2_ - receptor agonist that can continuously dilate the bronchi,^15^ while budesonide can alleviate airway inflammation and mucus secretion, and reduce airway obstruction factors. Therefore, combining these agents produces a synergistic effect, resulting in more significant improvements in FVC, FEV1%, and FEV1.^16^ The results of this study further support the observation by Zhao et al.^11^ which showed the considerable therapeutic effect of TIO combined with BUD/FORM on patients with moderate to severe COPD. This study confirms, therefore, that triple therapy performs better in improving lung function.^10^,^11^

Lung function improvement was shown to reduce symptoms and increase exercise tolerance in patients with COPD.^17^ Similarly, this study showed that compared to TIO monotherapy, the combination of TIO and BUD/FORM treatment resulted in decreased CAT scores and increased 6MWT in the elderly COPD cohort. A previous report by Welte et al.^18^ confirmed that the combination of budesonide/formoterol and TIO can significantly improve the course of the disease and health status of patients with COPD. This study further confirms that combination therapy improves lung function, alleviates symptoms such as respiratory distress, and enhances the patient’s exercise ability, which is imperative for improving the self-care ability and quality of life of elderly patients.^11^,^18^

HMGB1 and hs CRP are both inflammatory markers that reflect the severity of COPD. Extracellular HMGB1 can bind to corresponding receptors in the epithelium of the respiratory tract and small airways, mediating the secretion of chemokines and inflammatory cells and exacerbating airway inflammation.^8^,^19^ This study showed that after the treatment, the levels of HMGB1 and his CRP in the TIO+BUD/FORM group decreased more significantly compared to the TIO group, consistent with the results of Jiang et al.^19^ These observations strengthen the conclusion that the combination of TIO and BUD/FORM in the treatment of COPD can significantly reduce the patient’s inflammatory response, promote airway repair, and improve the disease course.

The evidence on adverse drug reactions in elderly patients with COPD undergoing multi-drug therapy is still scarce. Treating elderly patients with COPD presents unique challenges, such as declining physical function, weakened metabolic capacity,^20^ and a slower clearance rate of drugs that can easily lead to drug accumulation and adverse reactions.^21^ Elderly people often have multiple chronic diseases and a history of medication use, which may also increase the risk of adverse reactions.^22^,^23^ This study found that the incidence of adverse reactions was 8.8% (5/57) in the TIO+BUD/FORM group and 3.6% (2/57) in the TIO group, with no significant difference between the two groups. The main adverse reactions of combination therapy were constipation and dry mouth (two cases each), and palpitations (one case). A multicenter East Asian study by Lee et al.^24^ found that the incidence of adverse reactions in patients with severe and extremely severe COPD was 26% for both monotherapy and combination therapy, with no difference between the two groups. However, this study reported that while individuals with severe/extremely severe COPD comprised the majority of patients [70.2% (40/57)] in the group that received the combined therapy, the incidence of adverse reactions was lower (8.8%) than in the previous repo. Such discrepancy may be explained by the small sample size of this study. Further more extensive research with larger cohorts is needed to accurately evaluate the incidence of adverse effects associated with Tio monotherapy and the combined TIO+BUD/FORM regimens.

Although studies have demonstrated the effectiveness of TIO and BUD/FORM in treating COPD, data focusing solely on elderly patients are limited. Therefore, this study has important clinical implications. Based on the results of this report, for elderly patients with COPD, the combined TIO+BUD/FORM treatment plan should be preferred as it can yield additional benefits. This study provides new references for clinicians, which would help to optimize treatment strategies for elderly patients with COPD.

### Limitations:

This is a single-center retrospective analysis with a small sample size. Additionally, no long-term follow-up data were analyzed. Although improved lung capacity and exercise ability were observed in the TIO+BUD/FORM group, the impact on the quality of life may require longer treatment time. Additional higher-quality prospective studies are needed to validate the results of this study, and exacerbations in the elderly population also warrant further study.

## CONCLUSION

Compared with TIO monotherapy, combining TIO and BUD/FORM is more effective in treating elderly patients with COPD without increasing adverse reactions.

### Authors’ contributions:

**FS and DH**: Literature search, study design and manuscript writing.

**WL, XW, XL and TW**: Data collection, data analysis and interpretation. Critical Review.

**FS and DH:** Manuscript revision and validation. Critical analysis.

All authors have read, approved the final manuscript and are responsible for the integrity of the study.

## References

[1] Riley CM, Sciurba FC (2019). Diagnosis and Outpatient Management of Chronic Obstructive Pulmonary Disease:A Review. JAMA.

[2] Memon SI, Tanveer F, Usman G, Shakeel M (2024). Current practices in physiotherapy management of COPD in Pakistan. J Pak Med Assoc.

[3] Hu F, Lv F (2024). Effect of budesonide/glycopyrrolate/formoterol fumarate metered dose inhaler combined with nasal high-flow nasal cannula on elderly patients with COPD and respiratory failure. Pak J Med Sci.

[4] Kahnert K, Jörres RA, Behr J, Welte T (2023). The Diagnosis and Treatment of COPD and Its Comorbidities. Dtsch Arztebl Int.

[5] Xu H, Lu X (2019). Inhaled Glucocorticoid with or without Tiotropium Bromide for Asthma-Chronic Obstructive Pulmonary Disease Overlap Syndrome. J Coll Physicians Surg Pak.

[6] Anzueto A, Miravitlles M (2020). Tiotropium in chronic obstructive pulmonary disease - a review of clinical development. Respir Res.

[7] Lipworth B, Kuo CR, Stewart K, Chan R (2024). Budesonide/Formoterol or Budesonide/Albuterol as Anti-Inflammatory Reliever Therapy for Asthma. J Allergy Clin Immunol Pract.

[8] Huang J, Zeng T, Tian Y, Wu Y, Yu J, Pei Z (2019). Clinical significance of high-mobility group box-1 (HMGB1) in subjects with type 2 diabetes mellitus (T2DM) combined with chronic obstructive pulmonary disease (COPD). J Clin Lab Anal.

[9] Ellingsen J, Janson C, Bröms K, Hårdstedt M, Högman M, Lisspers K (2024). CRP, Fibrinogen, White Blood Cells, and Blood Cell Indices as Prognostic Biomarkers of Future COPD Exacerbation Frequency:The TIE Cohort Study. J Clin Med.

[10] Feng JF, Ding GR, Xie YZ, Zhao D, Wang X (2018). Efficacy of budesonide/formoterol and tiotropium combination for the treatment of Chinese patients with chronic obstructive pulmonary disease. Medicine (Baltimore).

[11] Zhao D, Ling C, Guo Q, Jin J, Xu H (2018). Efficacy and safety of tiotropium bromide combined with budesonide/formoterol in the treatment of moderate to severe chronic obstructive pulmonary disease. Exp Ther Med.

[12] Global Initiative for Chronic Obstructive Lung Disease (GOLD) (2025). Global strategy for the diagnosis, management, and prevention of chronic obstructive pulmonary disease:2025 report.

[13] CAT, COPD Assessment Test COPD Assessment Test.

[14] ATS Committee on Proficiency Standards for Clinical Pulmonary Function Laboratories (2002). ATS statement:guidelines for the six-minute walk test. Am J Respir Crit Care Med.

[15] Tashkin DP (2020). Formoterol for the Treatment of Chronic Obstructive Pulmonary Disease. Int J Chron Obstruct Pulmon Dis.

[16] Sun Y, Ren H, Han X, Yu S, An H, Yang X (2024). Clinical effect of qingre bawei capsules combined with budesonide in the treatment of acute exacerbation of chronic obstructive pulmonary disease. J Pak Med Assoc.

[17] Gulart AA, Munari AB, Santos Silva IJC, Alexandre HF, Karloh M, Mayer AF (2019). Baseline characteristics associated to improvement of patients with COPD in physical activity in daily life level after pulmonary rehabilitation. Respir Med.

[18] Welte T, Miravitlles M, Hernandez P, Eriksson G, Peterson S, Polanowski T (2009). Efficacy and tolerability of budesonide/formoterol added to tiotropium in patients with chronic obstructive pulmonary disease. Am J Respir Crit Care Med.

[19] Jiang T, Li P, Wang Y (2023). Effect of budesonide formoterol combined with tiotropium bromide on pulmonary function and inflammatory factors in patients with asthma-COPD overlap syndrome. Allergol Immunopathol (Madr).

[20] Fried TR, Vaz Fragoso CA, Rabow MW (2012). Caring for the older person with chronic obstructive pulmonary disease. JAMA.

[21] Matera MG, Hanania NA, Maniscalco M, Cazzola M (2023). Pharmacotherapies in Older Adults with COPD:Challenges and Opportunities. Drugs Aging.

[22] Crisafulli E, Sartori G, Vianello A, Busti F, Nobili A, Mannucci PM (2023). Clinical features and outcomes of elderly hospitalised patients with chronic obstructive pulmonary disease, heart failure or both. Intern Emerg Med.

[23] Bergs I, Just KS, Müller A, Stingl JC, Dreher M (2022). Emergency Department Visits Due to Dyspnea:Association with Inhalation Therapy in COPD and Cases with Adverse Drug Reactions. Int J Chron Obstruct Pulmon Dis.

[24] Lee HW, Park HM, Jang EJ, Lee CH (2022). Different inhaled corticosteroid doses in triple therapy for chronic obstructive pulmonary disease:systematic review and Bayesian network meta-analysis. Sci Rep.

